# Brugada Disease: Chronology Of Discovery And Paternity. Preliminary Observations And Historical Aspects

**Published:** 2003-10-01

**Authors:** Andres Ricardo Perez Riera, Edgardo Schapachnik, Celso Ferreira

**Affiliations:** * Associate Professor, ABC University, Santo Andre, SP, Brazil. In charge of the Electrovectorcardiography Area. Sao Paulo, Brazil; † Head of the Ambulatory Area for Chagas Disease, General Hospital of Acute Diseases Cosme Argerich, Buenos Aires, Argentina; ¶ Full Professor of Cardiology, ABC University, Santo Andre, SP, Sao Paulo, Brazil

**Keywords:** Brugada Disease, Brugada Syndrome, prehistory, history

## Abstract

The Brugada disease, the last clinico-cardiologic entity described in the 20th century, initially called right bundle branch block syndrome with ST segment elevation from V1 to V2 or V3 and sudden cardiac death, is genetically determined in a dominant autosomal mode, and it affects the alpha subunit of the Na^+^ channel by alteration of chromosome 3 and mutation in the SCN5A gene.

In clinical diagnosis the mentioned electrocardiographic pattern in a patient without structural heart disease and positivity in pharmacological tests are considered major criteria. As minor criteria, the following are considered: positive family history, presence of syncope with unknown origin, documented episode of VT/VF, inducibility in electrophysiologic study and positivity of genetic study.

The long-standing technology of ECG, with more than a century of existence, remains as the supplementary method with highest value in diagnosis, and currently new electrocardiographic criteria are suggested, which indicate high risk of VF.

Natural history indicates a somber diagnosis in symptomatic patients with a high index of arrhythmic SCD secondary to very fast polymorphic ventricular tachycardia bursts, which degenerate into VF. Asymptomatic individuals with only a Brugada-type electrocardiographic pattern have a low risk. The prognosis seems to depend more on clinical facts, since a positive electrophysiologic study has an accuracy of just around 50%.

We propose that this entity should be promoted to the category of disease, since it has a characteristic set of signs and symptoms, and an identified genetic defect.

## Preliminary Observations: "Prehistory"

Halfway through the 20th century (1953), Osher and Woff noticed the right bundle branch block (RBBB) electrocardiographic pattern, associated to ST segment elevation in the right precordial leads. These were considered at the time as normal variants, not having been related to sudden cardiac death (SCD) [1]. In 1975, Calo [2] reported an electrocardiographic triad that consisted of: R' wave, ST segment elevation and negative T wave in right precordial leads, which coincide with the characteristics of the electrocardiographic pattern currently known as Brugada-type, being considered a normal variant at the time.

During the 80s, the Center for Disease Control in Atlanta observed an abnormally high incidence of SCD in Asian refugees who immigrated to USA from the northeast of Thailand. This form of SCD is known in this country as Lai Tai (death during sleep). Approximately two decades later, the conclusion was reached that the entity known as "Sudden Unexplained Nocturnal Death Syndrome" (SUNDS) originates in an allele belonging to the same gene (SCN5A) as Brugada Disease [3].

In 1986, Prof. Pedro Brugada received his first patient with typical ECG, a Polish Caucasian child, who suffered several episodes of syncope. The boy presented as family background his sister's SCD, even though she had been treated with association of pacemaker implantation and amiodarone. In 1989, a patient with characteristic ECG was described as being a carrier of early repolarization syndrome [4]. In 1991, Pedro and Josep Brugada, adding 2 more cases, presented as an abstract in the NASPE meeting, a new clinical-cardiologic syndrome, typified by the association of RBBB, persistent ST segment elevation, normal QT interval and SCD [5].

## Initial Observations And Chronological Evolution: "History"

In 1992, the brothers from Catalonia (Spain), Pedro and Josep Brugada, presented the first description of the entity, adding four more patients to the initial description, making a total of eight [6]. This would be the last clinical-cardiologic entity to be identified in the 20th century [7]. One year later, Sumiyoshi et al [8] identified the syndrome described by the Brugada brothers as being a variant of idiopathic ventricular fibrillation (IVF) and in the same year, Proclamer et al [9] described a case, posing the possibility of a new arrhythmic syndrome with the characteristics described by the Brugada brothers. In the same year, Italian authors from different centers said that the alleged new entity is just a minor initial form or concealed form of right ventricle arrhythmogenic dysplasia/cardiomyopathy [10,11]. That same year, the Brugada brothers, in an Italian journal (G Ital Cardiol) replied to their colleagues from Padua and Nacarella, that they should not confuse issues, since in the new entity there is no underlying structural heart disease [12].

In 1994, Ferraccini et al [13] reported a case of IVF associated to RBBB and ST segment elevation, and in the same year, Bjerregaard et al [14] reported recurrent syncopes in a patient carrier of prominent J wave. In 1995, Tada et al [15] pointed out the significance of ST segment elevation in right precordial leads in patients with IVF, and Tohyou et al [16] analyzed the incidence of RBBB with ST segment elevation in normal population. In this same year, D'Onofrio et al [17] published an article wondering if the electrocardiographic pattern of RBBB associated to ST segment elevation from V1 to V3 in all the cases would correspond to a different syndrome.

In 1996, Gan-Xin Yan and Charles Antzelevitch [18], in an article where they approached the cellular basis of J wave in ECG, used the eponym Brugada for the first time to describe the syndrome discovered four years earlier. That same year, Kobayashi et al [19] called the typical electrocardiographic signs of the new entity as "Brugada type," and Miyazaki et al [20], pointing out to the autonomous modulation of ST segment elevation in patients with the syndrome described by the Brugada brothers, used the eponym just as Yan and Antzelevitch had done before. In the same year, Shimada also mentioned the eponym while reporting a case of the entity that presented monomorphic ventricular tachycardia (MVT) [21]. This is the first reference to a case of Brugada disease with MVT. In a short time, Dr. Bartolo Martini from the group of researchers from Padua claimed the paternity of the discovery, remarking that they had described the entity three years earlier, in 1989 [22]. In this work, the authors informed about the electrocardiographic manifestations as having a possible relationship with SCD; however, they concluded that in these cases there is a structural heart disease: right ventricle arrhythmogenic dysplasia/cardiomyopathy (RVAD), and did not acknowledge being in the presence of a new entity without an underlying organic substrate. Additionally, from the 6 patients mentioned in this article, only patient 3 presented the typical electrocardiographic pattern reported by the Brugada brothers [23,24].

In 1996, Corrado et al [25] presented the hypothesis of the existence of a subpopulation of RVAD that they called "concealed forms," which would present itself with the typical electrocardiographic features of this new entity proposed, including the presence of a polymorphic form of ventricular tachycardia. In the same year, such authors as Ohe and Fontaine followed the same line, which later was proved to be wrong [26,27]. Nevertheless, the alterations of the vago-sympathetic autonomous tone only constituted a triggering factor, but would not be the cause of the entity.

In 1997, Kobayashi et al [28] made the first description of the existence of an autonomous imbalance in Brugada disease, by means of the 123I-metaiodobenzylguanidine (MIBG) imaging techniques, which indicated the existence of a presynaptic autonomic dysfunction in the heart [29-31]. In 1997, Chinusi et al [32] made the first narration about the variable effect of disopiramide in ventricular arrhythmia induction in patients who are Brugada disease carriers, by acting on the Ito channel, sometimes increasing ST segment elevation, and possibly normalizing it.

In 1998, the acknowledgment of Brugada disease as an entity became unquestionable when Chen et al [33] identified three mutations in the SNC5A gene of chromosome 3p21-p24, which are responsible for Brugada disease and affect the alpha subunit of the Na+ channel. The mutations found were:

1. Missense or "wrong-information" mutation: this mutation affects exon 28, and in it, the amino acid glutamine is exchanged by leucine in codon 567 (L567Q) between domains I and II of the Na+ channel. This mutation determines a temporary increase in cation entrance during phase zero, with acceleration of recovery from the inactivation state.
2. Structural of frameshift mutation: which consists in the subtraction of a nucleotide in the SCN5A gene.
3. "Splice-donor" mutation: or mutation that accompanies the donor that affects intron 7 and introduces two bases of AA.

The last two cause a failure in the channel operation. In the same year, and for the first time, Nakamura et al [34] showed that the IC class antiarrhythmic agents, flecainide and pilsicainide, may cause ST segment elevation in the inferior wall. Later, it would be verified that ST segment elevation could manifest spontaneously in this wall in Brugada disease.

Makita et al [35] demonstrated the importance of the "overlooked" accessory beta 1 subunit of the Na+ channel. Thus, the authors explained that an alteration related to the alpha/beta subunits influences on the functional state of the Na+ channel, by causing a higher overlapping of activation and inactivation states, giving rise to a window current in T1620M and consequently, in VF triggering. Dr. Tagaki et al [36] described for the first time, employing an ultrafast computerized tomography, in 81% of a series of 26 patients, abnormalities in right ventricle wall motion, mostly located in the outflow tract (17 patients) or in inferior wall (4 patients), wondering about the functional nature of this entity.

## Year 1999

For the first time, Blazer et al [37] included Brugada disease within the chapter of ion channel diseases or channelopathies. The authors showed that the affected channels in Brugada disease are primarily, the fast Na+ channel, and secondarily, the initial K+ outflow channel or Ito channel or transient outward current in phase 1 or 4-aminipyridine-sensitive channel, and slow Ca2+ inflow channel in phase 2 or L-type ("L-type slow or long-lasting" calcium channel ICa-L type ICa2+- L). In this year, Bezzina et al [38] identified a single mutation in the Na+ channel, responsible for both Brugada disease and congenital long QT syndrome. In this way, the concept that both entities are allelic because they share the same locus was consolidated.

## Year 2000

In this year, Dr. Takanori Ikeda et al [39] identified the non-invasive markers of value in risk stratification in Brugada disease. The authors concluded that only high resolution ECG, and not QT interval dispersion or microvolt T wave alternans have value to identify the patients in high risk. They determined that high resolution ECG has a sensitivity of 89%, specificity of 50%, positive predictive value of 70%, and negative predictive value of 77% for the presence of late potentials (LP). The authors did not find a correlation between the degree of ST segment elevation and the HV interval. One year later, the same group of researchers confirmed in JACC the previous results [40]. Dr. Silvia Priori et al [41], in a prospective study of a numerous universe conducted with 52 families, arrived to the following conclusions:

1. asymptomatic individuals with Brugada-type electrocardiographic pattern, present a very low risk of SCD;
2. symptomatic with aborted SCD present a 23% of mortality rate in a mean 33-month follow-up;
3. the genetic mutation can be identified in a 15% of the cases;
4. the positive electrophysiologic study has a 50% of accuracy;
5. pharmacological tests have only a 35% of accuracy in asymptomatic carriers.

Nishizaki et al [42] showed the effect of insulin on ST segment elevation in this entity. 

## Year 2001

In May, Dr. Ihor Gussak and Dr. Hammill developed the major and minor criteria for diagnosing this entity [43]. The authors proposed that the presence of a major criterion and a minor one, constitute a diagnosis. They consider as major ones the electrocardiographic pattern in a patient without structural heart disease and positivity in pharmacological test; and as minor criteria the presence of positive family history, syncope of unknown origin, documented episode of VT/VF, inducibility in the electrophysiologic study, and positivity in genetic study (yet to be defined).

## Year 2002

In this year, genetic studies carried out by Vatta et al [44] showed that unexplained nocturnal SCD syndrome, known as SUNDS (Sudden Unexplained Nocturnal Death Syndrome) and Brugada disease are phenotypically, genetically and functionally identical and allelic since both affect the same gene: SCN5A. In November, the first virtual symposium about Brugada Syndrome was held (***VIRTUAL SYMPOSIUM ABOUT THE BRUGADA SYNDROME: TEN YEARS OF HISTORY: 1992/2002***)> http://www.brugada-symposium.org [45], which achieved an extraordinary worldwide impact, where relevant issues related to this entity were updated by specialists of international level, having as Honorary Presidents Pedro, Josep and Ramon Brugada; as President of the Scientific Committee Dr. Andres Ricardo Perez Riera; and as President of the Steering Committee Dr. Edgardo Schapachnik. The event approached basic research, genetics, supplementary methods for diagnosis, electrophysiology; it showed for the first time a vectorcardiogram of this entity; guidelines were discussed regarding sports practice and treatment.

The material available was organized in Lectures written by the Members of the Honorary Committee, and articles written by the most distinguished specialists in the area, reports of cases, and a round table broadcast through radio via Internet. It is currently possible to access the symposium on the Internet:  http://www.brugada-symposium.org

 During the event, a change of category was proposed for this entity: from syndrome to disease, founded in that it meets the criteria that define a disease, i.e.: all conditions that affect animals or plants, causing an interruption or modification of their performance as a response to environmental factors, specific agents, genetic defects, or the combination of these, and when at least two of these criteria are present:

1. known etiologic agent;
2. identified genetic defect;
3. set of characteristic signs and symptoms or compatible anatomic alterations, or
4. a combination of the previous items.

## Year 2003

Atarashi et al [46] suggested new electrocardiographic criteria that indicate high risk of VF in Brugada disease in a universe of 60 patients extracted from the Japanese register of Brugada syndrome. Thus, the authors established that:

1. S wave of duration = or > than 80msec in V1 has a predictive value of 40.5% and negative predictive value of 100% for VF with a 100% sensitivity;
2. ST segment elevation in V2 of 80msec or > measured from the J point has a positive predictive value of 37.8% and negative predictive value of 100% for VF with a 100% sensitivity.

Both criteria are highly specific indicators for VF in this entity.

## Conclusion

We go through "pre-history" and history since 1953, when Osher and Woff noticed an electrocardiographic pattern that resembled an acute myocardial lesion, which they attributed to a normal variant associated to RBBB and ST segment elevation in the right precordial leads. The historical steps followed to relate this pattern with SCD are described in people frequently in a productive age in life, predominantly in males, and without verifiable structural heart disease.

The original observation by the Spanish/Catalan Professor, Dr. Pedro Brugada in 1986, in a Pole caucasian and white child, with positive family background of SCD in a first-degree relative, ended in the initial description of the entity in 1992, called "A distinct clinical and electrocardiographic syndrome: right bundle branch block, persistent ST segment elevation with normal QT interval and sudden cardiac death." The impact of the discovery was so important, that in a few years the eponym: Brugada Syndrome, was employed nearly unanimously, by most investigators.
